# Clinical outcomes of screening and diagnostic mammography in a limited resource healthcare system

**DOI:** 10.1186/s12905-024-03007-0

**Published:** 2024-03-21

**Authors:** Mahmoud Al-Balas, Hamzeh Al-Balas, Zain AlAmer, Ghadeer Al-Taweel, Aseel Ghabboun, Farh Al Bzoor, Sumaia Abumkarab, Tala Abu Bakr, Batool Eleiwat

**Affiliations:** 1 Department of General Surgery, Urology and Anesthesia, The Hashemite University, Zarqa, 13133 Jordan; 2 Mu’tah University, Al-Karak, Jordan; 3 Department of Radiology, Ministry of Health, Amman, Jordan; 4 Hashemite University, Zarqa, 13133 Jordan; 5 Yarmouk University, Irbid, Jordan

**Keywords:** Breast cancer, Screening mammogram, Diagnostic mammogram, Screening recall, Screening recall rates

## Abstract

**Introduction:**

Breast cancer is a significant public health concern in Jordan. It is the most common cancer among Jordanian women. Despite its high incidence and advanced stage at time of diagnosis, the uptake of breast cancer screening in Jordan is low. This study aims to compare clinical outcomes of both screening and diagnostic mammogram among women in Jordan.

**Methods:**

A retrospective cohort of 1005 women who underwent mammography in breast imaging unit in a tertiary hospital in Jordan. It aimed to investigate outcomes of screening and diagnostic mammography. recall rates, clinical manifestations and cancer rates were investigated.

**Results:**

A total of 1005 participants were involved and divided into screening group (*n* = 634) and diagnostic group (*n* = 371). Women in the diagnostic group were more likely to be younger, premenopausal, smokers with higher BMI. Among the screening group, 22.3% were labeled with abnormal mammogram, 26% recalled for ultrasound, 46 patients underwent tissue biopsy and a total of 12 patients had a diagnosis of breast carcinoma. Among the diagnostic group, the most commonly reported symptoms were a feeling of breast mass, mastalgia and nipple discharge. Abnormal mammogram was reported in 50.4% of women, a complementary ultrasound was performed for 205 patients. A diagnostic Tru-cut biopsy for 144 patients and diagnostic excisional biopsy for 17 patients were performed. A total of 131 had a diagnosis of carcinoma.

**Conclusion:**

With the high possibility of identifying a carcinoma in mammography among symptomatic women and low uptake of screening mammogram, efforts to increase awareness and improve access to screening services are crucial in reducing the burden of breast cancer in Jordan.

## Introduction

Breast disorders, encompassing both benign and malignant conditions, are prevalent among women. While younger women more commonly experience benign diseases, breast cancer remains a significant cause of mortality in the female population at large [1, 2].

Mammography stands as the primary screening method for early breast cancer diagnosis due to its high sensitivity and specificity [3]. It also serves as a diagnostic tool for women presenting with symptoms [4]. Participation in mammographic screening has been associated with a substantial reduction (by 30%) in breast cancer mortality based on randomized trials of screening programs [5]. This efficacy is attributed to its ability to detect subtle architectural distortions and microcalcifications before a lesion becomes palpable [6].

However, despite its status as the gold standard, mammography can occasionally produce false negative results, especially in patients with dense breast tissue. Complementary imaging modalities, such as magnetic resonance imaging (MRI) and ultrasound, play a crucial role in improving sensitivity since dense breasts can obscure tumors [7, 8]. It’s essential to acknowledge that about 28% of cancers may be missed during screening mammography [9].

To evaluate the effectiveness of mammography practice, the term “medical audit” is used, involving the collection of patient results over a predefined time frame, typically one year. The audit is a valuable tool in assessing the detection of early-stage breast cancers while they remain treatable and identifying any gaps in technical performance and image interpretation. Consequently, the audit aids in reinforcing adherence to screening recommendations among patients and their referring healthcare professionals by providing compelling evidence of mammography’s effectiveness [10, 11].

During the audit, various rates are calculated, including the recall rate, which indicates the percentage of screened patients requiring further ultrasound or mammographic examinations. This rate is positively correlated with the likelihood of a false-positive result and is influenced by factors such as image quality and quantity, screening interval, single versus double reading, screening technique, and characteristics of the women being screened (e.g., age, use of hormonal therapy) [12, 13]​.

Women who are recalled for additional evaluation are at a higher likelihood of having breast cancer, particularly if they undergo a needle biopsy. As a result, such recalls can be distressing for patients [12].

## Materials and methods

This is a retrospective cross-sectional cohort study involving 1005 women who underwent mammography in the breast imaging unit at Prince Hamza Hospital, a tertiary referral institution located in the capital, Amman, during the period from June 2018 to February 2021. The study aimed to investigate the clinical outcomes of screening and diagnostic mammograms.

Data were collected from both electronic and paper-based medical records. It included patient information such as age, marital status, age of menarche, parity, age of the first child, menopausal age, smoking status, body mass index (BMI), indications for imaging, history of personal or familial breast cancer, and exposure to hormonal therapy.

Screening mammograms were defined as those performed in asymptomatic women who had no symptoms concurrent with the time of the mammographic examination. If symptoms were present, such as a breast or axillary mass, pain, nipple discharge or retraction, or skin changes, mammography was considered diagnostic.

Both mediolateral oblique and craniocaudal views were conducted for each breast. The interpretation of the mammographic images was carried out by two radiologists using The Breast Imaging Reporting and Data System (BI-RADS). Radiological findings, recall, and breast cancer rates were reported.

Data were analyzed using the Statistical Package for the Social Sciences version 23 (SPSS Inc., Chicago, IL) statistical software. The analyses included descriptive statistics and the chi-square test. A p-value < 0.05 was considered statistically significant.

## Results

A total of 1005 participants were included in our study, with a mean age of 52.4 (SD = 10). Women were classified into screening group (*n* = 634; 63%) and diagnostic group (*n* = 371; 37%). The age range of participants was between 29 and 89 years, with women in the diagnostic group being significantly younger than the screening group *P* = 0.02.

### Patients’ characteristics

Majority of women were married (93.7%) and covered by medical insurance (92.2%). The mean age of menarche was 13.6 years in screening group and 13.2 years in diagnostic group (*P* = 0.084). Women in screening group were more likely to be menopausal (59.8% vs. 48.9%; *P* = 0.001) with no significant statistical difference in the mean age of menopause between them.

The number and percentages of obese patients were 164.0 (25.9%) for screening group and 109 (33.9%) for diagnostic group; *P* < 0.001. Similar to obesity, women in diagnostic group were more likely to be smokers (25.2% vs. 18.5%) and less likely to have previous mammograms prior to their presentation with their symptoms (49.3% vs. 63.2%). More details can be seen in Table 1.

**Table 1:** Characteristics of the participants

Variables	Screening group (*N* = 634)	Diagnostic group (*N* = 371)	Total (*N* = 1005)	p value
**Age**				0.020^1^
Mean (SD)	52.9 (9.4)	51.4 (11.0)	52.4 (10.0)	
Range	33.0–81.0	29.0–89.0	29.0–89.0	
**Marital status**				0.225^2^
Single	35.0 (5.5%)	28.0 (7.5%)	63.0 (6.3%)	
Married	599.0 (94.5%)	343.0 (92.5%)	942.0 (93.7%)	
**Medical insurance**				1.00^2^
No	44.0 (6.9%)	25.0 (6.7%)	69.0 (6.9%)	
Yes	590.0 (92.9%)	346.0 (93.3%)	936.0 (93.1%)	
**Menarche**				0.084^1^
Mean (SD)	13.6 (4.3)	13.2 (1.6)	13.4 (3.6)	
Range	9.0–112.0	4.0–21.0	4.0–112.0	
**Menopause**				0.001^2^
No	255.0 (40.2%)	170.0 (45.8%)	425.0 (42.3%)	
Yes	379.0 (59.8%)	163.0 (44%)	542.0 (53.9%)	
Missed	0	38 (10.2%)	38 (3.8%)	
**Menopausal age**				0.059^1^
Mean (SD)	48.2 (4.8)	49.1 (5.2)	48.4 (4.9)	
Range	25.0–62.0	30.0–60.0	25.0–62.0	
**Parity**				0.423^2^
No	71.0 (11.2%)	49.0 (13.2%)	120 (11.9%)	
Yes	555.0 (87.5%)	304.0 (82.0%)	859 (85.5%)	
Unknown	8.0 (1.3%)	18.0 (4.8%)	26 (2.6%)	
**If previously pregnant, at what age first child was born**				0.054^1^
Mean (SD)	22.5 (5.1)	23.2 (5.5)	22.7 (5.2)	
Range	13.0–46.0	12.0–45.0	12.0–46.0	
**Smoker**				0.016^2^
No	427.0 (67.4%)	229.0 (61.7%)	656.0 (65.3%)	
Yes	117.0 (18.5%)	89.0 (24%)	206.0 (20.5%)	
Unknown	90.0 (14.2%)	53.0 (14.3%)	143.0 (14.2%)	
**BMI**				0.341
Non obese	202.0 (31.9%)	121.0 (32.6%)	323.0 (32.1%)	
Obese	164.0 (25.9%)	109.0 (29.4%)	273.0 (27.2%)	
Unknown	268.0 (42.3%)	141.0 (38%)	409.0 (40.7%)	
**Previous Mammogram**				< 0.001^2^
No	218.0 (34.4%)	170.0 (45.8%)	388.0 (38.6%)	
Yes	401.0 (63.2%)	169.0 (45.6%)	570.0 (56.7%)	
Unknown	15.0 (2.4%)	32.0 (8.6%)	47.0 (4.7%)	
**Personal history of breast cancer.**				0.203^2^
No	598.0 (94.3%)	328.0 (88.4%)	926.0 (92.1%)	
Yes	36.0 (5.7%)	13.0 (3.5%)	49.0 (4.9%)	
Unknown	0	30 (8.1%)	30 (3%)	
**Previous hormonal therapy**				0.243^2^
No	514.0 (81.1%)	268.0 (72.2%)	782.0 (77.8%)	
Yes	104.0 (16.4%)	53.0 (14.3%)	157.0 (15.6%)	
Unknown	16.0 (2.5%)	50 (13.5%)	66 (6.6%)	
**Family history of breast cancer**				0.703^2^
No	471.0 (74.3%)	262.0 (70.6%)	733.0 (72.9%)	
Yes	163.0 (25.7%)	96.0 (25.9%)	259.0 (25.8%)	
Unknown	0	13 (3.5%)	13 (1.3%)	

### Screening Group

Among the 634 women in the screening group, a total of 464 women (73.2%) were labelled as BIRAD 1 or BIRAD 2, 89 women (14%) as BIRAD 3 and, 51 (8%) as BIRAD 4, 2 women (0.3%) as BIRAD 5, and 28 women as BIRAD 0.

A total of 162 women who were labelled by mammogram as BIRAD 0, 3, 4 and 5 were recalled for breast ultrasound (26%). Among this group, 43 women (7%) underwent tru-cut core breast biopsy and 3 women underwent a diagnostic excisional biopsy. The results of core biopsy showed 10 patients (23.3%) had a diagnosis of cancer (DCIS, IDC, and ILC), while diagnostic excisional biopsy’s results revealed 2 patients with a diagnosis of carcinoma (Tables 2 and 3).

**Table 2:** Clinical outcomes of screening mammogram

Screening group	Overall (*N* = 634)
**Mammogram BI-RADs Score (** ***n*** ** = 634)**	
0	28 (4.4%)
1	108 (17.0%)
2	356 (56.2%)
3	89 (14.0%)
4	51 (8.0%)
5	2 (0.3%)
**Recall Breast Ultrasound (** ***n*** ** = 634)**	
No	462 (74.0%)
Yes	162 (26.0%)
Missing	10
**Core Breast Biopsy (** ***n*** ** = 634)**	
No	571 (93.0%)
Yes	43 (7.0%)
Missing	20
**Core Breast Biopsy Results (** ***n*** ** = 43)**	
Benign	33 (76.7%)
Malignant (DCIS, ILC, IDC)	10 (23.3%)
**Diagnostic Excisional Biopsy (** ***n*** ** = 3)**	
Benign	1
Malignant (DCIS, ILC, IDC)	2

**Table 3:** Core breast biopsy outcomes based on the screening BI-RADS

	BI-RAD 0	BI-RAD 1	BI-RAD 2	BI-RAD 3	BI-RAD 4	BI-RAD 5
**Number of core needle biopsies**	2	0	2	3	34	2
**Tissue biopsy results**						
** Fibroadenoma**	1	-	-	-	3	-
** Benign fibrocystic**	1	-	2	3	22	-
** Mastitis**	-	-	-	-	1	-
** DCIS**	-	-	-	-	4	-
** IDC**	-	-	-	-	3	2
** ILC**	-	-	-	-	1	-

### Diagnostic Group

Among women in the diagnostic group (*n* = 371), the majority of patients reported having more than one symptom. The most commonly reported symptoms were a feeling of breast mass or swelling (197; 53.1%), mastalgia (178; 48%) and nipple discharge (41; 11.1%) (Table 4).

**Table 4:** Clinical presentations among women refereed for diagnostic mammogram (*n* = 371)

Clinical presentation*	N (%)
Breast mass or swelling	197 (53.1%)
Mastalgia	178 (48%)
Nipple discharge	41 (11.1%)
Skin changes (ulcer or discoloration or thickening)	18 (4.9%)
Nipple changes (retraction, ulceration, inversion, eczema)	14 (3.8%)
Axillary mass	12 (3.2%)
Change in breast size	7 (1.9%)

*each woman reported one or more symptom based on her complaint

Regarding mammographic BIRAD score, 40 (10.8%) patients were labeled as BIRAD 1, 118 (31.8%) as BIRAD 2, 40 (10.8%) as BIRAD 3, 94 (25.3%) as BIRAD 4 and 53 patients (14.3%) as BIRAD 5. A complementary breast ultrasound was performed for 205 patients (57.4%) for further evaluation. A diagnostic Tru-cut biopsy was performed for 144 (40.5%) patients and 17 patients required diagnostic excisional biopsy. Among women who performed Tru-cut biopsy, the results of core biopsy showed 122 patients (84.7%) with diagnosis of breast cancer (DCIS, IDC, and ILC), while excisional biopsy’s results revealed additional 9 out of 17 patients with cancer diagnosis. A total of 131 (35.3%) of women in the diagnostic group had a diagnosis of breast carcinoma (Table 5).

**Table 5:** Clinical outcomes of diagnostic mammogram

Diagnostic group	Overall (*N* = 371)
Mammogram BI-RADs Score	
0	22 (6%)
1	40 (10.8%)
2	118 (31.8%)
3	40 (10.8%)
4	94 (25.3%)
5	53 (14.3%)
6	4 (1%)
**Breast Ultrasound**	
No	152 (41%)
Yes	205 (55.3%)
Missing	14
**Core Breast Biopsy**	
No	211 (57%)
Yes	144 (39%)
Missing	16
**Core Breast Biopsy Results (** ***n*** ** = 144)**	
Benign	19
Dysplasia	1
Intraductal papilloma	2
DCIS	12
IDC	93
ILC	17
**Diagnostic Excisional Biopsy**	17
Benign	8
Malignant (DCIS, ILC, IDC)	9

## Discussion

The incidence of breast cancer in lower- and middle-income countries (LMICs) has been on the rise, attributed to factors such as increased life expectancy, advancements in diagnostic modalities, the adoption of Western lifestyles, and changes in reproductive practices among young women [14, 15]. Notably, women in LMICs diagnosed with breast cancer tend to be younger, present at more advanced stages, and exhibit a higher prevalence of triple-negative cancers compared to higher-income countries [16, 17]. Consequently, there is a pressing need to focus on implementing breast cancer screening and early diagnosis strategies within the healthcare systems of LMICs.

Breast cancer screening strategies can be categorized as either population-based or opportunistic. In population-based screening, invitations for screening programs are issued from population-based registers, while in opportunistic screening, women decide to attend screening programs themselves or are referred by healthcare professionals.

Formerly, self-breast examination (SBE) and clinical breast examination (CBE) were part of screening modalities, but recent guidelines have excluded them due to their low prognostic yield [18–20]. However, it is worth noting that the utilization of SBE and CBE can still aid in identifying breast cancers in a majority of patients, especially in settings where mammography is not widely available as a population-based screening modality and can contribute to down-staging symptomatic disease ​ [18, 21]​.

The use of screening mammography has been associated with approximately a 20% reduction in breast cancer mortality, particularly among women aged 50 to 69 years [22]. Issues related to overdiagnosis and overtreatment among screened women, as well as its psychological burden, have been reported. Therefore, it is recommended to inform women about these issues prior to starting their screening [23, 24]. Achieving breast cancer screening goals requires the implementation of rigorous, high-quality screening, diagnosis, and treatment strategies.

In many LMICs, population-based mammogram screening is lacking, and opportunistic screening is the predominant practice. This can be attributed not only to insufficient infrastructure but also to a lack of qualified stakeholders involved in the screening process and the healthcare system’s capability to manage newly diagnosed cases and follow up on abnormal screening results ​ [25]​.

In Jordan, the Jordan Breast Cancer Program (JBCP), established in 2008, aims to increase breast cancer awareness among the Jordanian population and healthcare providers and implement strategies for opportunistic breast cancer screening and early diagnosis. According to the JBCP guidelines, average-risk women are advised to perform monthly SBE and CBE every 1–3 years until the age of 40. At 40, women are recommended to undergo breast mammograms every 1–2 years. Despite tremendous efforts by the JBCP in enhancing population knowledge, identifying possible barriers and conducting yearly awareness and educational campaigns, the uptake of breast cancer screening among Jordanian women is still low (7–9%) [26]. This low participation rate was comparable to that reported from Saudi Arabia, another Arabic country, which may reflect cultural barriers against screening for breast cancer [27, 28].

The utilization of breast ultrasound as an adjunct to screening mammogram improves screening outcomes, especially in women with dense breasts [29, 30]. In one Austrian study, adding breast ultrasound to mammography increased the cancer detection rate from 3.5/1000 to 4/1000 cases and from 1.8/1000 to 2.4/1000 among women with dense breasts [29]. The sensitivity of cancer detection in this study increased from 62% when mammogram is used alone to 81% when ultrasound is added to mammogram among dense breast women.

Among the screening group in our study, 26% of women underwent additional breast ultrasounds for further evaluation of their mammographic findings. While the recall rate set by the International Agency for Research on Cancer (IARC) for population-based screening is less than 7%, this figure may not be directly applicable to opportunistic screening settings. The use of supplementary ultrasound in this cohort is similar to that reported in an opportunistic screening group in China (31.5%) ​ [25].​.

Within the opportunistic screening group of this study, a total of 22.4% of screening mammograms were labeled as abnormal (BIRAD 3–5), and 8.4% were classified as suspicious (BIRAD 4–5). The recall rate among the screening cohort was 26%, the biopsy rate was 7.2%, and the positive predictive value (PPV) for biopsies was 26%. Notably, the cancer detection rate among this group was 19 per 1000 women, which is significantly higher than what has been reported in the literature [29, 30]. Reports from early breast cancer screening in Saudi Arabia and Egypt showed a cancer detection rate of 13% and 10%, respectively [27, 31].

## Conclusion

With the high possibility of identifying a carcinoma in mammography among symptomatic women and low uptake of screening mammogram, efforts to increase awareness and improve access to screening services can significantly enhance early detection and improve overall outcomes in breast cancer management.

## Data Availability

All datasets generated during the current study are not publicly available but are available from the corresponding author on request.

## Declarations

### Study limitations

A major limitation of this study is its single-center-based design, as well as its retrospective nature.
